# Does psychological ownership influence consumer happiness in playful consumption experience? Moderating role of consumer personality and game performance

**DOI:** 10.1016/j.heliyon.2023.e20236

**Published:** 2023-09-15

**Authors:** Muhammad Faisal Shahzad, Xie Ling, Jingbo Yuan

**Affiliations:** a School of Management, Shenzhen University China; b School of Medical Information Engineering, Zunyi Medical University, Zunyi, China

**Keywords:** Neuro Marketing,access-based consumption, Psychological ownership, Gaming, Emotions

## Abstract

This study examines the relationships between psychological ownership (PO) in playful consumption and its validating role in consumer happiness (CH). Specifically, we propose a moderating process of personality and game performance, which influences PO and CH. Subsequently, we evaluate consumer happiness associated with playful consumption experience by two studies, one quantitative and the other experimental. In Study 1 we use a randomized sample (n = 872, respondents) from Pakistan employing SEM (Structural Equation Modeling) methods. In Study 2 we use an EEG emotive insight device to capture the factors associated with psychological ownership. In this experiment, we address the neuro marketing prospect of players. It was found that perceived control, competitive resistance, emotions, customer participation, personality and performance are positively associated with PO. Game performance enhances feelings of happiness. This study offers new insights into the processes that drive consumer happiness and provides a vigorous guide for policymakers, applied psychologists, consumers and marketers who shape our futures in the field of happiness and well-being through playful consumption experience.

## 1 Introduction

Video games has gain lot more attention in developed and developing countries as its become an intimate aspect of the lives among millennial generation [1,2]. These days' young consumers reflect the increased autonomy towards online games and usually share their experience with friends about games [3]. It can be observed that playing online games is now become part of everyday leisure and socialization [4].

Consumer researchers have paid more attention to hedonic consumption, including aesthetic, symbolic, and expressive consumption. Studies identify some important but neglected aspects of consumption experience [5,6]. Hedonic consumption necessitates a brief examination considering consumer happiness, fun, moods and imaginations apart from everyday consumption [7]. Buying choices are not only associated with information acquisition, which usually pertains to purchasing decisions, but are also connected with ingenious, emotive, and approving consumption practices [8]. The difference between utilitarian and hedonic consumption is that in the latter individuals feel consumption as an enjoyable experience instead of simply buying, choosing or purchasing [9]. Studies have reported hedonic consumption experiences with products such as food, but the hedonic playful consumption experience has not been widely addressed [6]. To overcome this research gap, our study aims to understand the playful consumption experience, which is a combination of interconnected roles of sensations, performance, and temperament in the enjoyment of games [6]. Specifically, our purpose is to identify the factors that affect consumer psychological ownership in playful consumption of games (physical or online) and how consumers’ psychological ownership drives them to happiness and well-being. Moreover, the roles of personality and game performance are measured.

The present paper aims to address the research imbalance by focusing on hedonic consumption experiences, specifically on playful consumption illustrated by the interplay of emotions, performance, and personality in the enjoyment of games. This study aims to contribute to the growing body of research on experiential consumption and its impact on consumer behavior.

The study suggests that games provide a unique opportunity to examine the role of emotions, performance, and personality in shaping consumer enjoyment [3]. Games are an inherently playful activity that elicit a range of emotional responses and involve a clear performance metric, making them an ideal domain for studying the relationship between these factors. The study emphasizes that emotions play a crucial role in shaping the enjoyment of games. Positive emotions such as joy and excitement, as well as negative emotions such as frustration and anxiety, can both contribute to the overall experience of playing a game [10]. The study also highlights the importance of performance in shaping enjoyment, with players experiencing greater satisfaction when they perform well.

Finally, the study suggests that personality traits such as competitiveness, extraversion, and openness to experience can all play a role in shaping the enjoyment of games. Overall, the study provides valuable insights into the complex interplay between emotions, performance, and personality in shaping the enjoyment of hedonic consumption experiences.

TAM (Technology acceptance model) studies have largely reported consumer shifts towards psychological ownership and playful consumption experiences [11,12] however, there is need to explore its various dimensions in online games [2].

These days, playing video games is one of the most dominant and persuasive form of playful consumption among young consumers [6]. The Entertainment Software Association (ESA, 2019) reported that more than 214 million people in the U.S. play video games 1 h or more per week and 75% of all U.S. households have at least one person who plays. In sum, 64% of U.S. adults and 70% of those under 18 regularly play video games [13]. The market of video games is rapidly developing around the world, even in developing countries. Playing video games is a part of daily life. The video game industry has gained dramatic growth worldwide; for instance, in 2020, “the revenue from the worldwide PC gaming market was estimated at almost 37 billion U.S. dollars, while the mobile gaming market generated an estimated income of over 77 billion U.S. dollars” (ESA, 2021). Playing video games is more convenient these days; either you are playing online or offline by using your PC, smart phones, and other gaming devices. Abbasi, Ting [6]reported that playing video games is a hedonic experience in which consumers consider themselves involved in fun, imagination and pleasure. Consumers’ psychological possession in playing video games results in a fulfilling experience.

Psychological ownership satisfies intrinsic consumer motives, ensuring consumer happiness and well-being [14]. Psychological ownership triggers effective stimuli that can perceivecontrol reason and motivate themselves as a self-identity [15]. Apart from legal ownership, psychological ownership strengthens the relationship with the product and increases its perceived economic value [14,16]. The outcome of this kind of hedonic consumption (loyalty, satisfaction, increased demand) could be of great value for companies [17].

In this study, our objective is to provide a framework for 1) capturing factors associated with psychologicalownership, 2) linking consumer playful consumption behaviours with their happiness or well-being, and 3) understanding the role of game type in the relationship between psychological ownership and consumer happiness substantively. We seek to answer the following questions.

(1) Which factors are closely related to psychological ownership and what are their hypothesized relationships in an empirical study?
(2) What is the relationship between psychological ownership and consumer happiness in playful consumption experience?
(3) To what extent does personality and game performance affect the relationship between psychological ownership and consumer happiness?

To restate our contribution, our study is based on psychologicalownership theory, which proposes “an intimate connection between the individual and the target of ownership. A stronger association with the target [18] and a deeper knowledge about that target [4,19]create a preference for that target against other alternatives”. Prior studies [10] has revealed that psychologicalownership paves the path for consumer happiness. For instance studies on hospitality service experiences found that service experience endorse a sense of psychological ownership of the services they offered, which, in turn, develops customers' loyalty to service provider [20]. Danckwerts and Kenning [21]has worked on music‐based psychological ownership and found users' intention as an important factor to switch from free to premium consumption which result in enjoyable music consumption experience.

Therefore, our study aims to contribute by presenting and discussing the factors affecting consumer psychological ownership in playful consumption experience and how it can drive consumer happiness. First, in a survey setting, we aim to identify factors associated with psychological ownership followed by an experimental study to validate these results. In the next section we review literature and provide a conceptual framework.

## 2 Literature review

### 2.1 Playful consumption and consumer well being

Researchers have found that consumption is not just about buying tangible products. It is also connected to consumer happiness and well-being [6]. The world has witnessed a paradigm shift where the traditional model of buying or consuming has been digitalized. Consumers are now buying more digital resources in a networked economy context, ultimately making the buying experience more enjoyable [22]. Holbrook and Hirschman [23]highlighted the importance of hedonic consumption in the marketing domain. They found that buying behavior is not limited to purchasing choices but relates to enjoyable experiences such as “fun, feelings and fantasies”. Since then, hedonic consumption has been an important research topic in consumer buying behavior.

The term playful consumption is defined as “experience comprising imaginable, emotional, and sensory experiences to develop a scale to measure player experiences in video-game-playing” [6]. Playful consumption also refers to any activity where consumers seek pleasure, entertainment, sensory stimulation, excitement, and imagination [5]. Active consumption and consumer well-being in the context of video games remain less studied while they are innate parts of consumer personality [24]. These days playful consumption experience is growing due to the new form of playing facilities of video games and augmented or virtual reality gaming. For playful consumption of video games and happiness, the relationship is already established [22].

### 2.2 Antecedents of psychological ownership

Psychological ownership occurs when the consumer feels, emotionally speaking, that the product is “MINE.” The pursuit of consumer well-being has gained attention in consumer research in the recent past [25]. Pierce, Kostova [26]define psychological ownership as “that state in which individuals feels as though the target of ownership (material or nonmaterial in nature) or a piece of it is “theirs” (i.e. “It is MINE!)”. Two key dimensions of this technology-driven evolution of consumption reported by Ref. [27]are related to psychological ownership: “1) replacing legal ownership of private goods with legal access rights to goods and services owned and used by others, and (2) replacing “solid” material goods with “liquid” experiential goods”. Prior studies have attempted to understand the underlying mechanism of psychological ownership [15,28]. For instance, on experiences of ownership and their relation to music streaming formats found place, identity and perceived control as underlying motivations of psychological ownership and consumer happiness [29].

The psychological aspects in pursuit of consumer happiness have been established through many theoretical constructs such as self-achievement by social, escapism, word of mouth, willingness to pay more, customer company identification and competitive resistance [28,30]. Studies have often used the engagement construct in video storytelling builds relationship experiences to measure the relationship level [25]. Studies on video game playing by Ref. [16]have described achievement and escapism in virtual games as significantly associated with psychological ownership. Related constructs pertaining to game playing are stream, fascination, manifestation, and involvement [31]. Up to the present time, studies have identified constructs such asmotivations to certain objects, investing the self, pride and perceived control ling loyalty, empowerment and social rewards connected to psychological ownership [16,28,29,31]. Our study will also recognize distinct antecedents of psychological ownership, but what sets online and physical game players apart will be essential.

#### 2.2.1 Perceivedcontrol and psychological ownership

Perceived control in virtual gaming is a widely discussed construct. Studies have revealed that perceived control varies across a lifespan, during which perceived control is connected with a range of life domains such as achieving specific goals, enthusiasm, self-esteem, and mental/physical well-being [5]. Beliefs about perceived control commonly maintain that people dynamically manage their development. Specifically, individuals set goals, strive to achieve them and deal with costs over time. Perceived control is categorized into two groups, that is, primary and secondary perceived control. Primary perceived control occurs when individuals connect situations with their desires, whereas with secondary perceived control individuals set themselves according to the conditions. In this research, we have prompted psychological ownership as a primary perceived control variable in virtual games, consisting of goal-directed perseverance and determination [8]. In contrast, secondary perceived control includes a set of mechanisms, such as low success rates, accepting restrictions, and expecting benefits from a negative result [32].

Perceivecontrol in games has been found an important component of psychological ownership. Theory of planned behavior has reported in online games where the perceived behavioral perceived control is a direct predictor of behavioral intention [33,34].

Perceived control is a critical predictor of psychological ownership [16]. Research has evidenced that a low level of self-perceived control has a positive effect on online game addiction [35]. Primary–secondary perceived control has been widely studied in the field of education and healthcare [36] but as a construct it has received limited empirical attention in psychological ownership studies [37].studied the conceptualization of perceived control behavior and found that primary goals are linked with achievements and efforts, whereas secondary perceived control is an alternate approach when someone deals with an unfavorable outcome. It has been observed from studies that perceivedcontrol in online games.

Regardless of the theoretical application of primary and secondary perceived control to other fields, research is needed to recognize the effect of perceived control behavior on psychological ownership. In our study, we distinguish the role of perceived control in connection with the type of the game and how it influences consumer psychological ownership to achieve consumer well-being. Hypothesis 1 is formulated as follows.

H1 The perceivedcontrol in playing online gaming is positively associated with consumer psychological ownership.

#### 2.2.2 Emotions and psychological ownership

Playful consumption studies have uncovered different types of structure and functions but are less covered in the psychological ownership domain [8]. Consumer intrinsic drive measures certain behavioral aspects when engaged in sensational consumption activities such as playing video games [38]. Studies are less assertive in playful consumption, where gratification in consumption plays a pivotal role [39]. Achieving gratification in consumption is an individual's ability to use internal cognitive perceived control processes which in turn affect emotions in hedonic consumption [12,40]. The scheme proposed here is emotion regulation consumption (ERC), “which involves the consumption or purchase of a good or service for the purposes of alleviating, repairing, or managing an emotion in the short term” [39]. This paradigm can be used to examine emotion toward different types of play. Thus, the following hypothesis 2 is proposed.

H2 Emotions in playful consumption affect psychological ownership

H3 Psychological ownership has a positive effect on consumer happiness

### 2.3 Moderating role of personality and game performance

Consumer personality and game performance also affect consumer happiness. Personality variables may range from veracity in games to responsiveness and innovativeness or pleasure-seeking [41]. Personality traits are mainly relevant to the type of game a consumer is playing. Recent research has highlighted that consumer happiness related to consumption perspectives encompasses the game's kind [41]. From these interrelated perspectives, one assumption clearly emerging is that with different game preferences for physical versus online, these personality types show different emotional responses [42,43]. Therefore, we expected a moderating effect in the relationship of psychological ownership and consumer happiness on game type. Previously “personality-game congruity is established” as a fact of consumer emotions and playful consumption experience, but how it differs from game type would be an exciting avenue of research.

Game performance and emotional response are linked with game success. For instance, research has shown that players seeking high success rates tend to increase behaviorally assessed intrinsic impulse. Consumer happiness is hence perceived as a psychological drive. It can be observed from the literature that emotive responses in performance lead towards satisfying experiences [42,43].

In line with consumer psychology, intrinsic motivation to achieve better performance in games could gratify innate needs” [[43], [44], [45]]. Finally, we would expect current performance in playing will be positively associated with successive happiness. In addition to examining the relationship between psychological ownership and consumer happiness, our study examines the moderating role of personality and game performance in this relationship. The relationship between psychological ownership and consumer happiness is established especially among video and online games [13,22]but how other games can contribute to this relationship could be of significant interest. By making games more enjoyable and entertaining, consumer emotions of preference, excitement, and perceived control increase intrinsic impulse [6]. have identified playful consumption experiences in video games.

Therefore, we expect that.

H4 Personality moderates the relationship between psychological ownership and consumer happiness.

H5 Game Performance moderates the relationship between psychological ownership and consumer happiness.

**Fig. 1:**
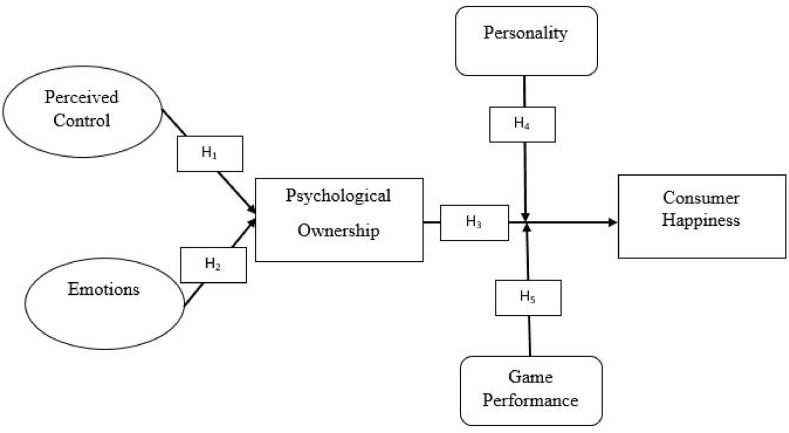
Conceptual model.

Fig. 1 (model) argues that “consumers first consume the game product in the form of “play” and get an experience of the game-play”. The concept of playful-consumption experience is derived from Ref. [46]. Moreover, consumer personality and game type is a moderating variable.

Self-determination theory addresses factors that either facilitate or undermine motivation, both intrinsic and extrinsic. In its early development the focus of SDT was on intrinsicmotivation, or motivation based in the inherent satisfactions derived from action [47]. According toSDT, intrinsic motivation is the core type of motivation underlying play and sport and clearly it is a type of motivation relevant to computergame participation for which, like sport, most players do notderive extra-game rewards or approval. Indeed, most computer game players pay to be involved, and some even facedisapproval for participating. Thus we suggest that peopletypically play these games because they are intrinsically satisfying as [48] puts it,because they are seeking “fun.”

## 3 Study 1

Study 1 utilized a quantitative approach by using a randomized sample to test the main proposition by identifying factors effecting psychological ownership and how it later drives consumer happiness. Study 1 also confirmed the moderating role of game type.

### 3.1 Methodology

A cross-sectional study design was employed for data collection from targeted participants [49]. This study design is widely used to acquire behavioral information such as beliefs and opinions. The targeted population for this study was generation Y and Z consumers in the age bracket of 6–39. The rationale behind selecting this cohort was their motivation to play games, either online or physically. An online questionnaire was designed to record the responses to enquire about their playful consumption experiences and happiness. Attaining consumer well-being through playful experiences could be an exciting phenomenon, especially among different types of game players. This methodology is in line with the study of [6], who measured teenaged consumers’ playful consumption and found that imagining and emotional experience are linked with cognitive, affective, and behavioral engagement.

### 3.2 Sample selection and size

An online version of the questionnaire was designed and circulated among the targeted cohorts; this approach was highly recommended in previous studies for greater reach. Consumers in these cohorts exhibit high acceptability of technology and media [26]. WhatsApp groups from different regions of Pakistan were recognized, and the e-version of the questionnaire was sent to record responses. Such data collection methods have been highly reported and recommended in previous studies to target consumer groups for data collection [50]. Convenience sampling was used to investigate response. According to Ref. [51]“the size of a convenience sample should be above or near 300 to prevent biases and errors”. The questionnaire was divided into two sections. Section one consisted of demographic details about gender, education, age and marital status, whereas section two consisted of study variables.

### 3.3 3Instrument and data collection

We developed a questionnaire by adopting items from prior literature; for instance, perceived control in gaming was measured with the statement “I insist on my right to explain what I want, until the others will understand me” (perceived control item 1), and another statement was “When I consider playing my own game I disregard marketing offers on other games” (Competitive resistance item 1). See appendix A for the study questionnaire. We used a seven-point Likert scale (from 1 = very unlikely to 7 = very likely) to record the feedback from consumers. The happiness associated with playful consumption was measured by eight items adopted from Ref. [52]. This scale was previously used to measure consumer well-being associated with restrained consumption behavior. This study investigated the reason behind sustainable anti-consumption, which includes voluntary simplicity, collaborative consumption, and debt-free living and its relation to consumer happiness or well-being. Then, we employed a three items scale measuring perceived control in gaming from Ref. [16]. The scale measured consumer psychological ownership, gaming motivation, and primary–secondary perceived control on online game addiction (OGA).

Next, we included a four-item scale for emotions in gaming, adapted from Ref. [46]. This scale has been widely used to measure experiential aspects of general consumption and intrinsically motivated hedonic enjoyment. The following five-item scale of psychological ownership was adopted from Ref. [17]. Consumer personality in gaming was measured with a ten-item scale adopted from Ref. [53]. This scale has been widely used to measure personality in 1 min or less. The last three-item scale measuring consumer game performance was adapted from Ref. [46]. In the second section, a question asked respondents whether they play games, eliciting a yes or no answer.

### 3.4 Demographics

Table 1 represents 59% respondents were male and 41% were female. The largest age group was 16–20 (29% of respondents), whereas 24% were 20–25 years old, with only 8% in the age bracket of 35–40 years old. Lastly, education levels showed diverse levels, with 38% at the bachelor degree level, 40% at the intermediate level and 21% at the master level.

**Table 1:** Respondents’ demographic characteristics.

VARIABLES	FREQUENCY	PERCENTAGE
GENDER		
Male	512	59
Female	360	41
**MARITAL STATUS**		
Married	259	31
Unmarried	613	69
**MY AGE**		
Less Than 15	8	1
16–20	251	29
21–25	212	24
26–30	196	22
31–35	142	16
36–40	63	8
**My family average monthly income is**		
>USD 50	0	0
USD50- USD100	26	3
USD101-USD150	34	4
USD151-USD300	96	11
<USD3000	716	82
**My Educational Level is**		
Matriculate	5	1
Intermediate	345	40
Graduate	318	38
Master and above	195	21

**Sample size:** 872 respondents.

### 3.5 Results

#### 3.5.1 Measurement model

For data analysis, SPSS 21.0 and AMOS were used. Indices such as comparative fit index (CFI), relative χ2 (CMIN/df), root mean square residual (RMR) and goodness of fit index (GFI) were used for data precision and model fitness. We obtained the result from specific indices (AGFI = 0.871, CFI = 0.92, GFI = 0.86, CMIN/df = 1.385, RMSEA = 0.062, RMR = 0.037 and TLI = 0.911) indicting model fitness based on data vigor. Convergent and discriminant validity was established for all given constructs. To test the reliability of given constructs, measures of composite reliability (CR) and the average variance extracted (AVE) values were measured. The threshold values for CR and AVE must be above 0.60 and 0.50, respectively [49]. In Table 2, standardized values of Cronbach's α reliability and CR are given [54]. The obtained values indicate decent internal consistency of items. The values of AVE indices also indicate a good fit and achieve the cut-off value of 0.5 and above at a significant level of p = .001 [55].methods were used to test the discriminant validity. Discriminant validity occurs when “two variables are predicted to be uncorrelated, and the scores obtained by measuring them are empirical” [49]. Discriminant validity results have shown in Table 3.

**Table 2:** Convergent validity results.

Measures	Factor loading	Cronbach's α	Composite reliability (CR)	AVE
Perceived control		0.721	0.812	0.614
When my friends think differently than me, I will convince them that I am right.	0.881			
I insist on my right to explain what I want, until the others will understand me.	0.914			
When I don't agree with others, I will try to convince them that I am right.	0.871			
	**Factor loading**	**Cronbach's α**	**Composite reliability (CR)**	**AVE**
**Emotions**		**0.845**	**0.798**	**0.891**
I don't pay much attention to my feelings when playing	0.913			
I usually care about what I'm feeling in game.	0.816			
One should never be guided by emotions in games.	0.917			
It is usually a waste of time to think about my emotions while playing.	0.828			
	**Factor loading**	**Cronbach's α**	**Composite reliability (CR)**	**AVE**
**Psychological Ownership**		**0.832**	**0.851**	**0.672**
I sense game is mine	0.852			
I feel personal ownership of this game	0.921			
I feel personally connected with this game	0.852			
It is hard for me to think about this game as mine	0.841			
It does not make me feel that it is mine	0.843			
	**Factor loading**	**Cronbach's α**	**Composite reliability (CR)**	**AVE**
**Personality**		**0.881**	**0.827**	**0.627**
I see myself in games as someone who is reserved	0.752			
I see myself in games as someone who is generally trusting	0.819			
I see myself in games as someone who tends to be lazy	0.873			
I see myself in games as someone who is relaxed, handles stress well	0.879			
I see myself in games as someone who has few artistic interests	0.819			
I see myself in games as someone who is outgoing, sociable	0.749			
I see myself in games as someone who tends to find fault with others	0.871			
I see myself in games as someone who does a thorough job	0.813			
I see myself in games as someone who gets nervous easily	0.911			
I see myself in games as someone who has an active imagination	0.878			
	**Factor loading**	**Cronbach's α**	**Composite reliability (CR)**	**AVE**
**Game Performance**		**0.845**	**0.73**	**0.584**
My game enables me to accomplish the purpose of playing game more quickly.	0.83			
My game enables me to fulfill the purpose of playing game effectively.	0.81			
My game enables me to satisfy the purpose of playing game easier	0.78			
	**Factor loading**	**Cronbach's α**	**Composite reliability (CR)**	**AVE**
**Consumer Happiness**		**0.812**	**0.814**	**0.694**
I lead a purposeful and meaningful life	0.815			
My social relationships are supportive and rewarding	0.875			
I am engaged and interested in my daily activities	0.797			
I actively contribute to the happiness and wellbeing of other	0.871			
I am competent and capable in the activities that are important to me	0.814			
I am a good person and live a good life	0.857			
I am optimistic about my future	0.819			
People respect me	0.831

**Table 3:** Discriminant validity results.

Factor	1	2	3	4	5	6
**Perceived control**	1 **(0.618)**					
**Emotions**	0.42	0.47	1 **(0.614)**			
**Personality**	0.45	0.52	0.49	1 **(0.704)**	0.47	
**Game Performance**	0.48	0.42	0.53	0.54	1 **(0.617)**	0.43
**Psychological Ownership**	0.41	0.42	0.48	0.41	0.43	1 **(0.708)**
**Consumer Happiness**	0.46	0.50	0.49	0.45	0.42	1

All correlations are significant at the p = .01, Square root AVE scores are displayed in parentheses.

Table 2 represents a significantly higher square root of AVE values of all constructs compared to their correlations with other constructs; therefore, results obtained from the measurement model test meet the required criteria. Based on the results of validity and reliability, measurement models were suitable to test the established propositions and structural models. We have utilized CFA (confirmatory factor analysis) methods using AMOS to test the reliability of the scales and their role in model fitness and testing given propositions.

#### 3.5.2 Hypothesis testing

**Fig. 2:**
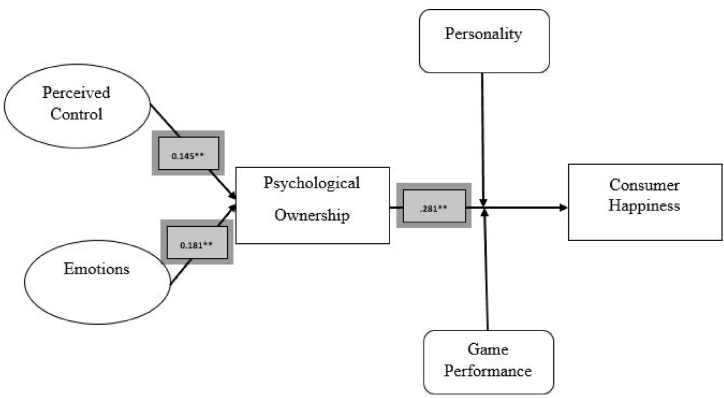
Structure Equation model path Coefficients.

Structural equation modelling (SEM) offered the essential instrument for measuring consumer happiness and how psychological ownership leads to consumer happiness. SEM is “a statistical modelling method of analysis that enables the testing of a series of separate, yet, interrelated constructs and regression equations, allowing for the analysis of multiple relationships at once” [40,56]. The indices used to evaluate the fitness of model were the CMIN/df, the GFI, the adjusted goodness of fit index (AGFI), the CFI, the Root Mean Square Error of Approximation (RMSEA), as well as the root mean residual (RMR) and the Tucker-Lewis index (TLI). The results of the given exhibited a good fit [57]. The obtained values were CMIN/df = 1.845, GFI = 0.87, AGFI = 0.851, CFI = 0.822, RMSEA = 0.061, RMR = 0.043 and TLI = 0.818. Path coefficients of the structural equation model are given in Fig. 2.

In order to accept hypotheses, the t-value, should be greater than 1.96 representing a significant path [58]. Examination of hypotheses presented a positive, significant relationship between perceived control in gaming and its impact on psychological ownership(β = 0.145, t = 2.524, p < .001), while a positive link was also found between competitive resistance and psychological ownership(β = 0.166, t = 3.232, p < .001). Similarly, consumer emotion in playing revealed a positive and significant effect on psychological ownership (β = 0.181, t = 2.672, p < .001), the effect of competitive resistance on psychological ownership was also found positively significant (β = 0.175, t = 3.419, p < .001), customer participation and its impact on psychological ownership was (β = 0.167, t = 2.122, p < .001), the effect of personality on psychological ownership was (β = 0.152, t = 2.301, p < .001), game performance and psychological ownership were also significantly related (β = 0.192, t = 3.122, p < .001), thereby supporting Hypotheses 1–7, respectively. Finally, psychological ownership showed a significant positive influence on consumer happiness in playful consumption experience (β = .281, t = 4.245, p < .001), thus confirming Hypothesis 8.

#### 3.5.3 Moderation of personality

**Fig. 3:**
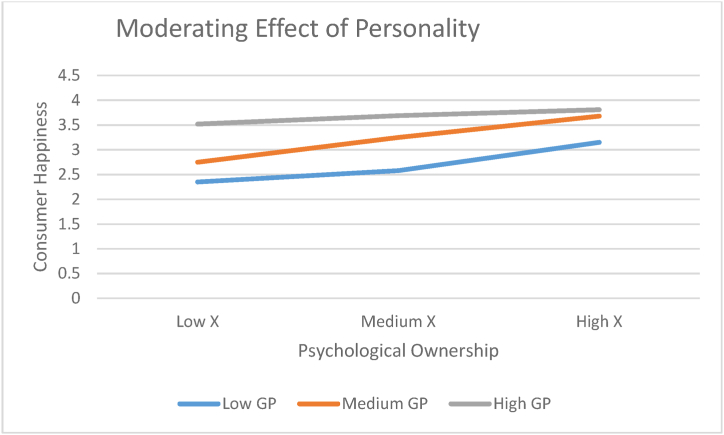
A plot of Psychological Ownership (PO) on Consumer Happiness (CH) versus the moderator (Personality) with Playful consumption experience.

Process macro, Model 1 [36] using SPSS 21.0 was applied to test moderating effects. The following statistics were R^2^ = 0.17 F (2, 8145 4.92, p = .001). Results showed a significant PO*GT interaction CH (R2-chng = 0.01; b = 0.01, F = 3.91, p = .05). The values of the moderated indirect and direct effects of GT in influencing consumer happiness (CH) (H9). Results have shown Low and medium levels of PO have a significant positive indirect effect (low) = 0.83, 95% CI. = 0.24, 1.42; and indirect effect (medium) = 0.57, 95% CI = 0.17, 0.94. On the other hand, for higher values of GT, there is an insignificant relation (indirect effect [high] = 0.29, 95% CI = −0.24, 0.85) for H9. The conditional indirect effect is positive but declines as the GT increases. Fig. 3 shows the indirect and direct effect (psychological ownership and consumer happiness) at varying levels of GT with a 95% confidence interval. The result of Fig. 3 suggests that the indirect effect between psychological ownership and consumer happiness is conditional upon the level of GT. At a certain level of GT, consumer happiness motives decrease. The Johnson-Neyman technique results are shown in Table 4 [56,59,60] Results suggest that the relationship between psychological ownership and consumer happiness are significant only up to a certain level (i.e., .62), beyond which this relationship becomes insignificant.

**Table 4:** Conditional effect for different values of the moderator (P) using the Johnson-Neyman technique.

P	Effect	se	t	p	LLCI	ULCI
*−1.59*	*1.03*	*0.47*	*2.52*	*0.02*	*0.15*	*1.98*
*−1.43*	*0.99*	*0.44*	*2.13*	*0*	*0.17*	*1.87*
*−1.27*	*0.93*	*0.4*	*2.57*	*0.02*	*0.19*	*1.78*
*−1.1*	*0.9*	*0.37*	*2.14*	*0.03*	*0.21*	*1.68*
*−0.94*	*0.87*	*0.34*	*2.98*	*0.01*	*0.22*	*1.63*
*−0.78*	*0.82*	*0.32*	*2.64*	*0.01*	*0.23*	*1.53*
*−0.62*	*0.78*	*0.29*	*2.57*	*0.02*	*0.23*	*1.47*
*−0.45*	*0.73*	*0.27*	*2.47*	*0.04*	*0.22*	*1.38*
*−0.29*	*0.68*	*0.25*	*2.45*	*0.01*	*0.21*	*1.29*
*−0.13*	*0.64*	*0.23*	*2.17*	*0.01*	*0.2*	*1.21*
*0.03*	*0.59*	*0.22*	*2.17*	*0.01*	*0.17*	*1.12*
*0.14*	*0.55*	*0.2*	*2.95*	*0.02*	*0.13*	*1.05*
*0.3*	*0.5*	*0.2*	*2.14*	*0.01*	*0.08*	*0.98*
*0.46*	*0.46*	*0.23*	*2.15*	*0.01*	*0.05*	*0.93*
*0.54*	*0.44*	*0.23*	*2.02*	*0.02*	*0*	*0.9*
0.62	*0.41*	*0.24*	*1.98*	*0.05*	*0.01*	*0.87*
0.79	0.37	0.26	1.72	0.13	−0.1	0.86
0.95	0.32	0.28	1.64	0.17	−0.21	0.86
1.11	0.28	0.31	1.21	0.21	−0.37	0.85
1.27	0.23	0.34	1.01	0.38	−0.41	0.85
1.44	0.19	0.37	0.82	0.51	−0.52	0.88
1.6	0.14	0.4	0.49	0.54	−0.68	0.91

**Notes**: To investigate the interaction of Psychological Ownership and Game Type on Consumer Happiness MACRO incorporating the Johnson-Neyman technique was utilized, using arbitrary points of the moderator (i.e. P). The results reveal ranges of the moderator in which the focal predictor (PO) is a significant predictor of the outcome (i.e. CH). Highlighted values in italic indicate that the conditional effect was a significant predictor of CH.

#### 3.5.4 Moderation of game performance

The following statistics were R^2^ = 0.19 F (3, 1472 4.97, p = .001) were obtained. Results showed a significant PO*GP interaction CH (R2-chng = 0.01; b = 0.01, F = 4.73, p = .05). The values of the moderated indirect and direct effects of GP in influencing consumer happiness (CH) (H5). Results revealed low and medium levels of PO have a significant positive indirect effect (low) = 0.81, 95% CI. = 0.25, 1.41; and indirect effect (medium) = 0.57, 95% CI = 0.33, 0.98. On the other hand, for higher values of GT, there is an insignificant relation (indirect effect [high] = 0.24, 95% CI = −0.22, 0.87) for H5. The conditional indirect effect is positive but declines as the GP increases. Fig. 3 shows the indirect and direct effect (psychological ownership and consumer happiness) at varying levels of GP with a 95% confidence interval. The result of Fig. 3 suggests that the indirect effect between psychological ownership and consumer happiness is conditional upon the level of GP. At a certain level of GP, consumer happiness motives decrease. The Johnson-Neyman technique results are shown in Table 5 [56,59]. Results suggest that the relationship between psychological ownership and consumer happiness are significant only up to a certain level (i.e., .48), beyond which this relationship becomes insignificant.

**Table 5:** Conditional effect for different values of the moderator (GP) using the Johnson-Neyman technique.

P	Effect	se	t	p	LLCI	ULCI
*−1.74*	*1.01*	*0.51*	*3.74*	*0.03*	*0.18*	*2.154*
*−1.58*	*0.96*	*0.48*	*3.49*	*0.02*	*0.16*	*1.95*
*−1.42*	*0.9*	*0.45*	*2.46*	*0.03*	*0.14*	*1.67*
*−1.25*	*0.87*	*0.39*	*2.57*	*0.02*	*0.15*	*1.52*
*−1.09*	*0.85*	*0.37*	*2.69*	*0.02*	*0.18*	*1.47*
*−0.92*	*0.81*	*0.31*	*2.74*	*0.02*	*0.21*	*1.34*
*−0.76*	*0.77*	*0.29*	*2.86*	*0.04*	*0.2*	*1.29*
*−0.6*	*0.72*	*0.28*	*2.98*	*0.03*	*0.2*	*1.15*
*−0.44*	*0.69*	*0.24*	*2.96*	*0.01*	*0.2*	*1.12*
*−0.28*	*0.68*	*0.21*	*2.95*	*0.01*	*0.19*	*0.99*
*−0.17*	*0.62*	*0.19*	*2.89*	*0.02*	*0.18*	*95*
*−0.01*	*0.57*	*0.18*	*2.75*	*0.01*	*0.16*	*0.9*
*0.15*	*0.51*	*0.18*	*2.54*	*0.01*	*0.06*	*0.88*
*0.23*	*0.48*	*0.18*	*2.21*	*0.01*	*0*	*0.85*
*0.4*	*0.45*	*0.18*	*2.11*	*0.05*	*−0.01*	*0.84*
*0.48*	*0.41*	*0.17*	*1.95*	*0.05*	*−0.1*	*0.82*
0.64	0.37	0.21	1.65	0.15	−0.19	0.82
0.8	0.32	0.22	1.18	0.18	−0.26	0.84
0.96	0.29	0.22	0.95	0.21	−0.34	0.86
1.13	0.25	0.29	0.67	0.25	−0.49	0.86
1.29	0.18	0.35	0.58	0.36	−0.58	0.88
1.45	0.13	0.31	0.39	0.57	−0.64	0.89

**Notes**: To investigate the interaction of Psychological Ownership and Game Type on Consumer Happiness MACRO incorporating the Johnson-Neyman technique was utilized, using arbitrary points of the moderator (i.e. P). The results reveal ranges of the moderator in which the focal predictor (PO) is a significant predictor of the outcome (i.e. CH). Highlighted values in italic indicate that the conditional effect was a significant predictor of CH.

### 3.6 Discussion - study 1

This study has examined the phenomenon of psychological ownership based on the constructs of perceived control, emotions, personality and performance to better predict consumer psychological ownership in games and its validating role in consumer happiness [8]. The results showed that the factors mentioned above affect psychological ownership. Consumers spend a lot of time playing online games at this age due to their enjoyment of games, emotions, performance, and personality [46]. The availability of smartphones has made it easier for youth to spend a lot of time playing online games and become active players [16]. However, the modern consumer's lifestyle induces playing online [42]. The study has found a positive effect of perceived control in gaming on psychological ownership. This means consumers who have more power in gaming can experience more ownership of their game.

Moreover, the study highlights the positive impact of game performance on psychological ownership. Companies that are product ordinated can influence consumers’ perceptions of games, whereby competitive offers can enrich consumer predisposition to specific games [61]. Interestingly, emotion in games can encourage ownership by inducing players to consider themselves as a virtual identity [62]. Furthermore, consumer personality excites emotions by focusing on performance and achievement, which not only provides ownership but also provokes happiness [42]. Furthermore, the proliferation of these factors in different games can create ownership, consequently tempting enjoyment.

On the other hand, it was found that psychological ownership endorses positive feelings that arouse happiness [30]. Moreover, this study indicates that perceived control, emotions, personality and performance in games may breed positive emotions that stimulate psychological ownership. In a similar vein,Li and Atkinson [8] found that consumer happiness is linked with increased psychological ownership through mediation of basicpsychological needs. It has also been observed that factors associated with playful consumption experience and its validating role with psychological ownership can yield pleasure stimuli [8]. These positive moods can trigger satisfaction with such consumption, especially among young consumers [8].

This study establishes that consumer personality and game performance moderates and strengthens the relationship between psychological ownership and consumer happiness [63]. Findings suggest that consumers are more likely to continue playing a game that they perceive to fit their personality [41]. Research on online gaming has revealed that consumers’ virtual identities help them achieve their playing goals [42]. Therefore, consumer game type becomes the foundation of achieving happiness in gaming [16].

## 4 Study 2 EEG measurements

In this experiment subject was seated at ease on chair in a softly lit soundproof room and an EEG electrode cap was placed on their head. Subjects were requested to minimize head and body movements during recording. Two standard laptops were used and subjects were asked to play game of their choice at stage one. Only one game can be played in one time and subjects were instructed to think about the feelings they are experiencing during play. Appendix I depicts the visual presentation of EEG recording.

The objective of experimental Study 2 is to test the factors associated with consumer psychological ownership in playful consumption experience using a neuromarketing approach.

### 4.1 Design and method

Our objective was to record EEG emotive insight response of players while playing video games and interprets the emotions for different types of games [64]. The EEG test was conducted in a noise proof room with adequate required light settings. Sample of young consumers aged 18 to 26, were tested using EEG emotive insight [65]. Those who usually play video games were selected. Two computers were arranged for this experiment for game playing and EEG recording separately. Players were asked to put the EEG emotive device on their head and after ensuring the best quality signals, the experiment was performed.

Steenkamp and Baumgartner [57]has conducted an experiment using EEG with those consumers who have responded to a questionnaire in relation to their excitement and joy towards commercial adds. Results reported that theta and alpha band activities were related to the subsequent pleasantness ratings, with activity in the left frontal cortex related to “pleasant” commercials and activity in the right frontal cortex associated with “unpleasant” commercials. This experiment reveals behavioral response during EEG recording and might not be correlated with the actual valuation that could be limitations of the study.

Games included in this experiment were PUB G, Scorer and Need for Speed (car racing). These games include different game characters such as Victor, Carlo, Sara and Andy, famous charterers of the PUB G game, where consumers perceive themselves as virtual players or as part of that team. After putting on the EEG emotive, players were asked to stay still and calm in order to register EEG readings for different phases. We collected players’ sensation data using EEG emotive. The factors included in these sensations were perceived control, emotions, personality, and game performance.

### 4.2 Results and discussion

Results have shown through EEG Emotive that emotions for the game and personality are related to game type. The assessment of the emotion readings was done on the basis of average psychological ownership about the game. Average values and variance, as well as the percentage of time when psychological ownership level was high (above 0.8) are shown in Table 6. Originally, we also calculated these values for the Emotive headset. At every stage of experiment the averaged values of players were taken. Our experiment consisted of both non-engaging mentally and as well as highly engaging parts [66]. Duo (group with other friend) and squad (group with up to three friends) was non-engaging, whereas solo active game playing required high mental involvement (Pub G).

**Table 6:** Average values and variance.

	Stimuli's	Avg. PO	PO var.	Avg. H	H Var.	TPO	TH
1	Pub G Solo	0.452	0.175	0.581	0.07	14%	4%
2	Pub G Duo	0.450	0.183	0.541	0.128	18%	5%
3	Pub G Squad	0.501	0.225	0.532	0.174	22%	9%
4	Victor	0.417	0.206	0.517	0.153	17%	8%
5	Andy	0.571	0.190	0.491	0.161	23%	4%
6	Sara	0.593	0.218	0.546	0.153	21%	6%
7	Carlo	0.540	0.215	0.514	0.164	25%	7%

PO = psychological ownership, H=Happiness, TPO = Total psychological ownership, TH = total happiness, TPO - % time psychological ownership >0.8; TH - % time Happiness >0.8.

**Fig. 4:**
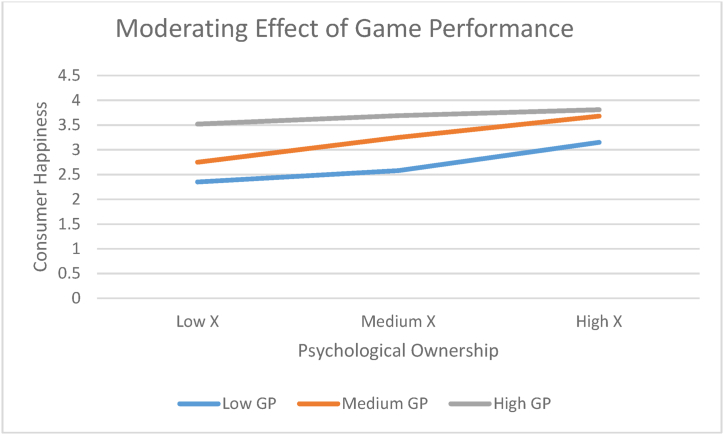
A plot of Psychological Ownership (PO) on Consumer Happiness (CH) versus the moderator Game Performance (GP) with Playful consumption experience.

Psychological ownership results showed that average psychological ownership and the time of high psychological ownership when playing with other persons were higher than when playing “Solo”. For “Duo” results are somewhat similar, but variance is lower. Happiness remained approximately constant when playing “Solo”, but variance increased when playing Pub G “Squad”, compared to playing “Duo”. Happiness results for non-engaging activities were higher. The highest involvement was obtained when playing “Sara” (a game character who is a vehicle expert and loves automobiles and going for rides) as most of the players agree with this character. Psychological ownership when playing Pub G match and happiness values calculated for various electrodes are shown in Fig. 4. Results shown in the graph representing only slight game perceived control changes can be observed between the 170th and 240th seconds (emotions in seconds while playing the Pub G game calculated using Emotive software). On the other hand, rapid change can be seen during customer participation as players were very highly involved at this point.

The psychological ownership graph shows related changes, but psychological ownership like competitive resistance does not evoke ownership in this graph. In the final part both relationship intentions and performance correspond to amazingly high ownership at the 182nd and 354th seconds of the game. The performance and personality levels were averagely recorded in these experiments at the 342nd and 354th seconds.

**Fig. 5:**
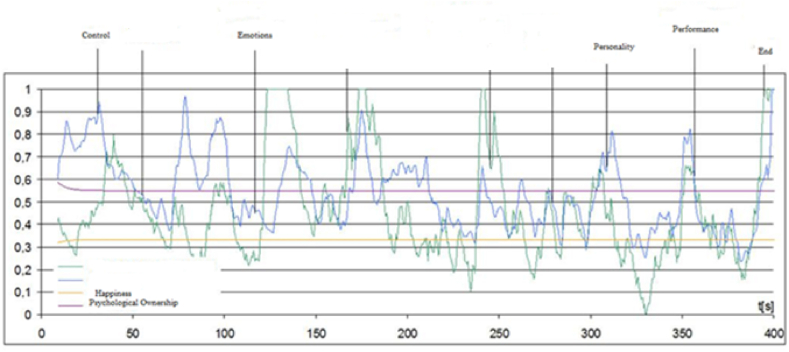
Psychological ownership during the Pub G match, calculated using Emotive EEG software.

This graph in Fig. 5 represents the Happiness graph. For instance, only slight emotion changes can be observed between the 170th and 240th seconds when playing the game.

Based on the findings, this research has three important theoretical and practical implications. First, this study identifies factors affecting psychological ownership in the context of Pakistan. Secondly, it provides a practical guide to policy makers who are seeking consumer happiness linked with playful consumption experience. Lastly, our study finds game type to be an important construct in Pakistan due to increased virtual game playing experience. For example, virtual game companies can offer more connected experiences to customers to gain a high market share. Drawing from psychological ownership theory we identify perceived control, competitive resistance, emotions, customer participation, personality and performance as important factors in playful consumption experience [29]. In developing counties such as Pakistan, the usage of smartphones has increased due to the expansion of the mobile and IT industry, along with an accumulation of physical gaming, which endorses the importance of well-being associated with playful consumption experience [8]. Our study is in line with [65]who have worked on players' neurophysiology when playing a game. Similar interpretations have been made in other studies, where researchers have observed changes in the power of brainwaves when players are highly involved in the context of the game [24]. In sum, we find that factors such as perceived control, competitive resistance, emotions, personality and performance that reflect an individual's behavior are in line with previous research. Our results reveal that in order to identify related emotions during gameplay, EEG data should be processed efficiently [67].

## 5 Conclusions

Study 1 investigated factors affecting psychological ownership, and their validating role in consumer happiness. The study also identified moderating role of personality and game performance in this relationship. It was found that playful consumption experience paves the way for consumer happiness. Results specify the positive role of perceived control, competitive resistance, emotions, personality and performance to better predict consumer psychological ownership in games and its validating role in consumer happiness. These constructs breed emotional affections and emotions that generate happiness, while P & GP moderates these emotions. Our experimental study identifies the phenomenon of psychological ownership in video game playing and its impact on consumer happiness. We utilized the neuromarketing EEG method for advanced outcomes. We found that playing a game is associated with brain activities changing and evoking emotions when playing a game. Study 2 highlights important contributions for academic practitioners. The term psychological ownership has been recognized in extended literature, but in this study we have investigated it in a new way while using the EEG technique. Specifically, these results contribute to the study of consumer playful consumption experience, which focuses on how consumers achieve happiness. Gaming companies can utilize these techniques to gain real insights into the gameplay experience, which would help marketing research. The EEG emotive can detect emotions related to gameplay but these recordings are not publicly available so precise information could vary. This study like any other study has some limitations. First, we address the broad context of gaming, that is, the online and the physical. However, consumer happiness may vary according to the type of game. For instance, these days consumers like to play virtual games more frequently. Future research could examine the difference between game players; for instance, whether psychological ownership is greater when playing online or physically. Similarly, the role of gender could provide new insights. In our study, gender is treated as a perceived control variable. Lastly, results from other countries and cultures that differ from Pakistan could provide novel findings. In our results of study 2 we found low variability of perceived control in gaming and performance whereas emotions and personality had a large variability. Such inconsistencies have also been shown in similar work with EEG emotive.

Emotions play an important role in the enjoyment of games, as they can enhance or detract from the overall experience. Positive emotions such as joy and excitement can contribute to a sense of flow and immersion in the game, while negative emotions such as frustration and anger can disrupt the experience and reduce enjoyment [12]. Performance is another key factor in the enjoyment of games, as players often derive satisfaction from achieving goals, overcoming challenges, and improving their skills [46]. However, excessive pressure to perform or a lack of challenge can also detract from the experience. Overall, by examining these facets of the consumption experience, the study provides insights into the experiential aspects of playful consumption and highlights the importance of emotions, performance, and personality in the enjoyment of games [27].

The distinction between using and choosing a product highlights the importance of the experience of using the product, as opposed to just the act of selecting it. The experience of using a product can be influenced by a variety of factors such as the design, functionality, and user interface, which can impact consumer satisfaction and loyalty [14]. Overall, by emphasizing the experiential side of these distinctions, we gain a more holistic understanding of consumer behavior that takes into account not just the practical aspects of buying and using products, but also the emotional and experiential dimensions that shape consumer preferences and behaviors [27].

This paper emphasizes the importance of game motivations in understanding the development of psychological ownership and its potential relationship to gaming. Specifically, individuals who are motivated by achievement and escapism are more likely to develop a sense of psychological ownership towards the virtual gaming world, which may lead to gaming addiction if taken to extreme levels. Additionally, the paper discusses the role of primary and secondary control in online gaming addiction. Primary control refers to individuals who seek to control their environment, while secondary control refers to individuals who adapt to their environment. Those who exhibit high levels of primary control may be more prone to gaming addiction because they feel a need to control their virtual environment. Overall, the paper highlights the importance of understanding game motivations and the potential for psychological ownership in the development of gaming. It also raises awareness of the role of primary and secondary control and emphasizes the need for individuals to make informed decisions regarding their gaming activity.

## Limtions and future research

The study have found that there is no prdective power of precison. Future studies sought to overcome these limitations and can validate the role of EEG in conumers studies among some different devloped countries. Oldder segemnt of consumers would be more intresting.

The limitations can be overcome by using different stimuli and then recording emotions. Thus, more profound experiments may provide more exciting results. Future studies can investigate emotion time in different intervals of game play. The study of [3] has also stressed this factor that must be explored in future. Addressing factors causing psychological ownership in playful consumption empirically makes important contributions to existing research on consumer happiness and well-being.

## Funding statement

This paper was financially supported by the National Natural Science Foundation of China（Grant Number：72172094）; Humanities and Social Sciences Foundation of the Ministry of Education in China（Grant Number：21YJC630160 and 2018 Special Project for Cultivation and Innovation of New Academic，Qian Platform Talent [2018]5772－012.

## Data availability statement

Data will be available on request.

## Ethics approval (include appropriate approvals or waivers)

Ethical approval to conduct the current study wasobtained from University of Management and Technology (UMT's) institutional review board (IRB) following Helsinki protocols. Written informed consent publish the survey reported data were obtained from the respondent. The present study adheres to the principles of the Helsinki Accord and was approved by the institutional ethical review committee (UMTREC 324).

## Author contribution statement

Muhammad Faisal Shahzad; Ling Xie: Conceived and designed the experiment; Performed the experiments; Analyzed and interpreted the data; Wrote the paper.

Jingbo Yaun: Contributed reagents, materials, analysis tools or data.

## Declaration of competing interest

The authors declare that they have no known competing financial interests or personal relationships that could have appeared to influence the work reported in this paper.

## Figures and Tables

**Figure:**
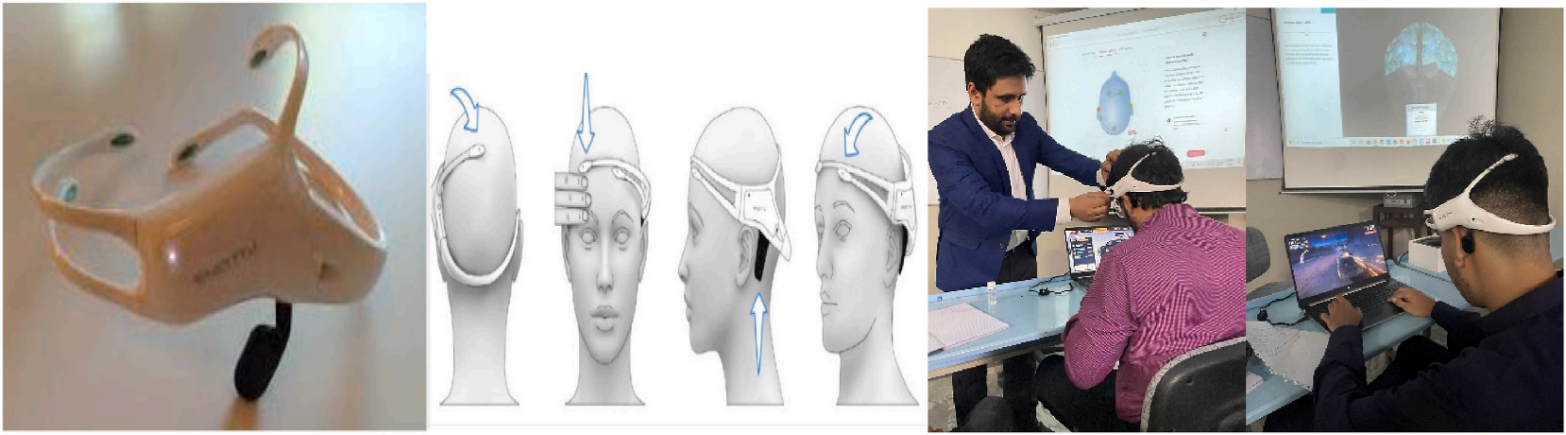
Emotive Insight EEG
