# Corrigendum to “Does psychological ownership influence consumer happiness in playful consumption experience? Moderating role of consumer personality and game performance” [Heliyon Volume 9, Issue 9, September 2023, article e20236]

**DOI:** 10.1016/j.heliyon.2025.e43356

**Published:** 2025-05-24

**Authors:** Muhammad Faisal Shahzad, Xie Ling, Jingbo Yuan

**Affiliations:** a School of Management, Shenzhen University, China; b School of Medical Information Engineering, Zunyi Medical University, Zunyi, China

In the original published version of this article, declarations related to parental consent and consent for publication of images were missing.

The original declaration showed as below:

**Ethics approval (include appropriate approvals or waivers)**

Ethical approval to conduct the current study was obtained from University of Management and Technology (UMT's) institutional review board (IRB) following Helsinki protocols. Written informed consent publish the survey reported data were obtained from the respondent. The present study adheres to the principles of the Helsinki Accord and was approved by the institutional ethical review committee (UMTREC 324).

The missing declaration have now been added in the correct version which can be found below:

**Ethics declarations**

Ethical approval to conduct the current study was obtained from University of Management and Technology (UMT's) institutional review board (IRB) following Helsinki protocols; approval number UMTREC 324. Written informed consent to participate and publish the survey reported data were obtained from respondents. Additionally, for minor participants, written consent of parents was sought before allowing them to participate. Lastly, consent for publication was sought from participants for the images included in the article.

The authors apologize for the errors.

## Declaration of competing interest

The authors declare that they have no known competing financial interests or personal relationships that could have appeared to influence the work reported in this paper.

